# QGWFQS: A Queue-Group-Based Weight Fair Queueing Scheduler on FPGA

**DOI:** 10.3390/mi14112100

**Published:** 2023-11-14

**Authors:** Yunfei Guo, Zhichuan Guo, Xiaoyong Song, Mangu Song

**Affiliations:** 1 National Network New Media Engineering Research Center, Institute of Acoustics, Chinese Academy of Sciences, No. 21, North Fourth Ring Road, Haidian District, Beijing 100190, China; guoyf@dsp.ac.cn (Y.G.); songxy@dsp.ac.cn (X.S.); songmg@dsp.ac.cn (M.S.); 2 School of Electronic, Electrical and Communication Engineering, University of Chinese Academy of Sciences, No. 19(A), Yuquan Road, Shijingshan District, Beijing 100049, China; 3 Suzhou Haiwang Network Technologies Co., Ltd., Suzhou 215163, China

**Keywords:** FPGA, WFQ, queue scheduler, queue group

## Abstract

Weight Fair Queuing is an ideal scheduling algorithm to guarantee the bandwidth of different queues according to their configured Weights when the switching nodes of the network are congested. Many of the switching nodes based on FPGA in the current network support four physical ports or hundreds of virtual ports. Massive logic and storage resources would be consumed if each port implemented a WFQ scheduler. This paper proposes a Queue-Group-Based WFQ Scheduler (QGWFQS), which can support WFQ scheduling across multiple ports through the reuse of tag calculation and encoding circuits. We also propose a novel finish tag calculation algorithm to accommodate the variation in the link rate of each port. The remainder of integer division is also taken into account, which makes the bandwidth allocation fairer. Experimental results show that the proposed scheduler supports up to 512 ports, with 32 queues allocated on each individual port. The scheduler has the capability to operate at 200 MHz and the total scheduling capacity reaches 200 Mpps.

## 1. Introduction

### 1.1. Motivation

Network providers require efficient allocation and management of bandwidth resources for network links [1,2]. Different flows are assigned to distinct queues, and the switch schedule packets from these queues use specific scheduling algorithms. Commonly employed scheduling algorithms in switches include Strict Priority, Weight Round Robin, Deficit Round Robin [3], Weight Deficit Round Robin, and Weight Fair Queuing. Scheduling algorithms like WDRR and WFQ, which consider packet length during scheduling, are well-suited for bandwidth resource allocation. They eliminate the need for prior knowledge of the average packet length across different queues and ensure that bandwidth allocation remains unaffected by variations in the average packet length [4]. However, WDRR encounters challenges [5] when servicing a large number of queues, resulting in excessively long service cycles. If the number of active queues changes within a service cycle, whether the scheduler initiates a new scheduling cycle or continues with the ongoing cycle, the scheduling results will deviate from the desired outcome. WFQ is an ideal scheduling algorithm for bandwidth resource allocation, as it guarantees the minimum bandwidth for each queue during congestion.

However, implementing WFQ algorithms in FPGA poses significant challenges due to the limited availability of memory and logic resources. Most FPGA-based switches typically support only two or four physical ports, while SmartNICs based on FPGA are capable of serving hundreds of virtual machines on a server, with each machine corresponding to a virtual port. Whether it is a physical or virtual port, each port constitutes a queue group. The number of queue groups is equivalent to the total number of ports, and each group requires an independent WFQ scheduling logic. However, current approaches face difficulty in calculating packet tags and sorting them across multiple queue groups without each group having its own WFQ scheduler replication. This replication would result in a substantial consumption of logic and storage resources.

### 1.2. Limitations of Prior Art

The current research in FPGA-based implementation of the WFQ scheduling algorithm primarily focuses on the development of circuits to compute the finish tags of packets and subsequently sort these computed tags.

McKillen [6] proposed an FPGA implementation of the WFQ scheduling algorithm, which utilizes a pipeline approach to calculate the finish tag for each incoming packet, even if they originate from the same queue. However, this computational approach introduces redundancy requirements on the maximum sorting capacity supported by the sorting circuit, as it transmits the tags of sequentially arriving packets from the same queue to the sorting circuit. In order to achieve a desirable level of network bandwidth management, a switch deployed within a data center is typically required to support the WFQ scheduling algorithm for a range of 24 to 32 queues per queue group [7]. However, the number of queue groups requiring the implementation of the WFQ scheduling algorithm may potentially surpass several hundred or even exceed this magnitude. In such a scenario, the existing method for calculating finish tags is deemed inadequate, as the computed tags are confined within a singular scheduling logic. Furthermore, in the process of calculating packet tags, this algorithm relies solely on the reciprocal of the sum of Weights of all backlogged queues as the slope for the virtual time increment. The obtained virtual time is considered accurate only when the overall link rate of the queue group remains constant, as elaborated in Section 2.2. Consequently, scenarios where the link rate of each queue group can change dynamically are not accommodated. In addition, the existing algorithm performs two division operations when calculating the virtual time and tag for a specific packet. It utilizes the sum of Weights of all backlogged queues as the divisor for the virtual time calculation, and the Weight of the corresponding queue as the divisor for the tag calculation. However, only the quotient obtained through integer division is utilized in subsequent computations, while the remainder is not taken into consideration.

The existing sorting methods can be categorized as register-based [8] and BRAM-based [9,10], depending on the storage location of the tags to be sorted. Despite the advantages of BRAM-based sorting methods, such as their ability to handle a larger number of tags and run at higher frequencies compared with register-based methods, these approaches still impose a substantial demand on storage resources, even in straightforward scenarios involving the scheduling of 32 queues. While implementing such sorting circuits on a single queue group necessitates a relatively modest allocation of storage resources, the cumulative resource consumption becomes significantly greater than the available resources of FPGA when sequentially implementing them across hundreds of queue groups.

Therefore, due to the aforementioned considerations regarding algorithmic and resource constraints, it becomes infeasible to achieve the implementation of a scheduler within an FPGA that can simultaneously handle multiple queue groups, each containing a scheduling logic responsible for servicing 24 to 32 WFQ queues.

### 1.3. Proposed Approach

To facilitate the implementation of independent WFQ algorithms on multiple queue groups, we introduce a novel queue-group-based WFQ scheduler comprising a tag calculation module and a queue scheduling module. The scheduler collaborates with a Shared Packet Buffer and a packet classify and distribute module. The innovations of our proposed scheduler include:

- Less Resource Consumption. Only the packet at the head of each WFQ queue is eligible for potential scheduling. When the head packet of a queue is scheduled and there are remaining packets in the same queue, the new head packet of that queue is then subjected to tag calculation and potential scheduling. With this Queue Head Packet Scheduling Model (QHPSM), when selecting a queue for scheduling from a queue group, the number of tags to be compared is reduced to no more than the maximum number of backlogged queues within the queue group. This implies that it is unnecessary to maintain tag sorting within each queue group continuously. Once a new tag is computed, it is temporarily stored in a buffer. When queue selection for scheduling is required, the tags corresponding to the head packets of all backlogged queues in the queue group are compared, and the minimum tag is determined. This comparison circuitry can be reused for each queue group, which results in the scheduler occupying only a minimal amount of resources.
- Support Variable Port Link Rate. The schedule algorithm does not care about the correspondence between virtual time and real time, and the growth rate of virtual time remains unaffected by the link rate. The scheduler solely requires the detection of port idleness and the presence of a queue in need of scheduling, which is easy to implement in FPGA.
- Fairness. During each division calculation, the remainder is considered. The generated remainder from the tag calculation of the last packet within the same queue is recorded, and this previously recorded remainder is utilized to compensate and rectify the tag calculation for subsequent packets in the queue. This approach enhances the fairness of the implemented scheduling algorithm.

## 2. Related Work

### 2.1. Tag Sorting Circuit

McLaughlin [11] demonstrated an efficient hardware sorting circuit and subsequently achieved fully implementation [12] of the WFQ algorithm on FPGA. During the sorting process, a linked list structure is employed to store the sorted tags, while a lookup tree and a translation table are utilized to determine the position of new tags within the linked list. This sorting methodology enables the retrieval of the packet with the lowest value within a fixed and predictable lookup time.

PIFO [8] employs a mechanism whereby elements are pushed into their respective positions within a priority queue based on their priority level. When elements are to be scheduled, PIFO selects and dequeues them from the front of the priority queue. However, PIFO lacks scalability due to the significant requirement for parallel comparisons.

PIEO [9] uses BRAM instead of registers to store sorted tags, which significantly improves the scalability of the sorting circuit, and can run to a higher main frequency, thereby improving scheduling capabilities.

Gearbox [10] achieves Approximate WFQ by replacing sorting circuits with a packet scheduler composed of a relatively small number of FIFOs. This approach allows for occasional minor sorting deviations while introducing a small amount of additional unfairness.

### 2.2. Tag Calculation Method

Generalized Processor Sharing (GPS) [13] is an ideal bandwidth allocation algorithm that treats the packets from different backlogged queues as fluid and concurrently schedules them bit by bit. In a single scheduling instance, the number of bits scheduled for each queue is proportional to its Weight. We assume that at time *t*, a queue group, which can represent either a physical port or a virtual port, has a bandwidth capacity of C(t) and there are *n* backlogged queues with Weights $w_{1}$, $w_{2}$, …, $w_{n}$ in this queue group. The bandwidth allocated to each backlogged queue $r_{i}$ can be expressed as Equation (1).

(1) $$r_{i}=\frac{w_{i}}{\sum w_{i}}C\left(t\right),\;\mathrm{for}\;i=1,2,\ldots ,n,$$

In practice, messages are transmitted over networks in the form of packets, making packets the smallest schedulable unit. Consequently, the GPS scheduling algorithm is not directly applicable to real-world networks. The Weighted Fair Queuing (WFQ) [14] scheduling algorithm approximates the GPS scheduling algorithm from an implementable perspective by simulating the calculation of the start time and finish time of each packet in the GPS scheduling algorithm. It selects the packet with the minimum finish time for scheduling. We denote the *j*th packet of queue *i* as $p_{i}^{j}$, the packet length of $p_{i}^{j}$ as $l_{i}^{j}$, and the arriving time of $p_{i}^{j}$ as $A(p_{i}^{j})$. Then, the calculation algorithm of the start time $S(p_{i}^{j})$ and the finish time $F(p_{i}^{j})$ of $p_{i}^{j}$ is found from Equations (2) and (3).

(2) $$S\left(p_{i}^{j}\right)=max\left\{v\left(A\left(p_{i}^{j}\right)\right),F\left(p_{i}^{j-1}\right)\right\},j\geq 1$$

(3) $$F\left(p_{i}^{j}\right)=\left\{\begin{array}{cc} S\left(p_{i}^{j}\right)+\frac{l_{i}^{j}}{w_{i}} & ,\;\mathrm{if}\;j\geq 1\\ 0 & ,\;\mathrm{if}\;j=0 \end{array}\right.,$$

where v(t) represents the virtual time utilized to simulate the temporal aspect of an ideal GPS scheduler. The virtual time is governed by the fulfillment of Equation (4).

(4) $$\frac{dv(t)}{dt}=\frac{C(t)}{\sum w_{i}},\;\mathrm{for}\;i=1,2,\ldots ,n,$$

The growth rate of v(t) is directly proportional to C(t) and inversely proportional to the sum of Weights of all backlogged queues. For a queue group, the total bandwidth resources may not necessarily remain constant. Therefore, accurately computing v(t) in hardware requires obtaining precise values of C(t) at any given moment, which is challenging in FPGA due to the discrete nature of time and transmitted bits. The packet tag calculation method, proposed by McKillen [6], based on WFQ assumes that C(t) is constant, which can adversely affect the accuracy of v(t), $S(p_{i}^{j})$, and $F(p_{i}^{j})$ calculations when C(t) changes, thereby impacting scheduling fairness.

The Self Clock Fair Queue (SCFQ) [15] scheduling algorithm is based on the WFQ algorithm and simplifies the virtual time update algorithm. The algorithm of SCFQ to calculate the start time and end time of service packets is the same as that of WFQ, but the algorithm of calculating virtual time is updated as Equation (5).

(5) $$v\left(t\right)=F_{l}^{k},\;s_{l}^{k}<t\leq d_{l}^{k},$$

where $s_{l}^{k}$ and $d_{l}^{k}$ represent the start service time and end service time of the packet currently departing the scheduler. The SCFQ scheduling algorithm considers that during the period when a packet is scheduled, the finish time of the packet is updated as the virtual time of the current system. The virtual time computed by the SCFQ scheduling algorithm no longer represents the time within the hypothetical GPS system but serves merely as an indication of the current progress of the scheduler. Updating the virtual time directly using the values calculated during the algorithmic computations, rather than defining it as a function of link rate C(t) and the sum of Weights of backlogged queues, reduces the complexity of the scheduler algorithm design.

SCFQ seems to be a fair queue scheduling algorithm suitable for deployment on hardware circuits [16]. However, for the calculation of completion time, SCFQ adopts the algorithm of WFQ without specific optimizations for hardware implementation, which may introduce scheduling unfairness. High-precision floating-point division operations can be easily performed in software, but they pose challenges in hardware. A method for performing integer division in hardware [17] is proposed, which calculates the quotient and remainder resulting from the division of two integers. In the SCFQ algorithm implemented based on integer division, the accumulation of remainders can affect the accuracy of the finish tag calculation, thereby impacting the fairness of scheduling outcomes.

## 3. Proposed Scheduler

In the subsequent discussion, the maximum number of queue groups supported by our work is denoted as *M*. The maximum number of fair queues supported by each queue group is uniformly defined and denoted as *N*.

**Figure 1:**
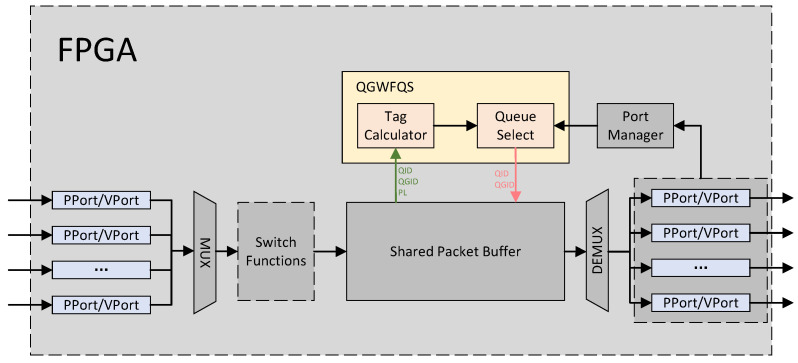
Overall architecture. PPort: physical port. VPort: virtual port.

The overall architecture of the proposed Queue-Group-based WFQ Scheduler, consisting of Tag Calculator and Queue Select, is shown in Figure 1.

Packets arriving from various physical or virtual ports are merged into a single stream. Then, they are processed using Switch Functions [18,19] such as parsing, matching, action execution, and deparsing. Subsequently, the packets with attributes such as Queue Group ID (QGID), Queue ID (QID), and Packet Length (PL) are cached in a Shared Packet Buffer (SPB). SPB enqueues the attributes of the head packet of the queue into QGWFQS, as indicated by the green arrow in the diagram, when one of the following conditions is met. Condition one occurs when a packet arrives at an empty queue. Condition two arises when the queue is still non-empty after the head packet of the queue has been dequeued.

The Port Manager monitors the status of each port and transmits the QGID of the idle port requiring scheduling to QGWFQS. QGWFQS selects an appropriate queue for scheduling from the queue group and sends the QID and QGID of the scheduled queue to SPB via the pink arrows in the diagram. The packet at the head of the corresponding queue then departs from SPB and is finally transmitted through the respective port.

### 3.1. Shared Packet Buffer

The input and output packet interface of SPB is AXI-Stream Interface. The attributes of the incoming packets, such as QGID, QID, and PL, are carried by the USER signal of AXI-Stream Interface. Before a packet arrives at SPB, its QGID and QID are determined through one or several Match-Actions during the Switch Functions stage. The PL is also parsed by the parser during the Switch Functions stage.

**Figure 2:**
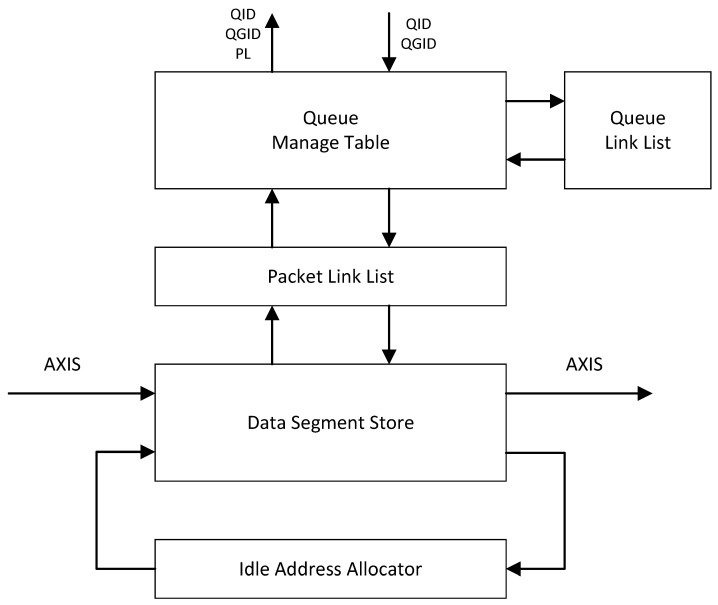
Structure of Shared Packet Buffer.

The structure of SPB is shown in Figure 2. Each part of SPB is well pipelined. The Data Segment Store (DSS) is the place where the data of the packet are cached. The DSS is divided into *l* number of segments, each of which has the same size of $B_{size}$. Queue Manage Table (QMT), together with Packet Link List (PLL) and Queue Link List (QLL), abstract these segments into m∗n number of FIFOs, each of which can be mapped to a queue.

Once a packet arrives at SPB, it is firstly cut into one or several Data Blocks (DBs) according to $B_{size}$. The first DB of the packet is defined as First Data Block (FDB). If the packet length is less than $B_{size}$, the only DB is regarded as FDB, obviously.

All the addresses of the idle DSS segments are cached at the Idle Address Allocator (IAA). The IAA is an FIFO with a depth of *l*. During system initialization, all of the *l* number of DSS segments addresses are pushed into the IAA as idle addresses. Each time a DB is writing into the DSS, an idle address is pulled from the IAA. And the segment, which this address is pointing to, is the place where the DB will be written into. Similarly, each time a DB has been read out from the DSS, the address of the segment where the DB was cached will be pushed into the IAA as an idle address.

All of the addresses of the DBs from the same packet are cached in the form of a singly linked list in the PLL, which is a memory with *l* number of addresses. The content that is stored at each address in the PLL is Next Data Block Address (NDBA) and PL. Once the PLL obtains the address of the FDB of a packet, each DB’s address of this packet can be read out sequentially. After the DB has been written into the DSS, the address of DB is sent to the PLL. The PLL always caches the address of the DB received last time. Then, if this time the receiving address does not belong to an FDB, this receiving address is written as NDBA at the PLL’s cached address, which is the address of the DB received last time.

For example, a packet is cut into three DBs according to its packet length, and the three DBs are denoted as DB1, DB2, and DB3. DB1 is written into address *A*, DB2 is written into address *B*, and DB3 is written into address *C*. Then *A*, *B*, and *C* will be sent to PLL. *A* first, then *B*, and finally *C*. When the PLL receives *A*, it does nothing except caching it because *A* belongs to an FDB. Then, the PLL receives *B*, and *B* is written as NDBA at the address of *A* in the PLL. The packet length of this packet is also written as PL at the same place. At the same time, the PLL deletes the cached *A* and then caches *B*. After a while, the PLL receives *C*. Similarly, *C* is written as NDBA at the address of *B* in the PLL. As for reading packets, the PLL receives *A*, the address of the packet’s FDB, from the Queue Manage Table. The content of the address of *A* stored in the PLL is *B* and PL. The PLL then can easily read *B* and *C* out, and then finally send all the address of DBs and the PL of the packet to the DSS. The DSS then reads out the data of the packet and reassembles this packet.

Based on the function of the PLL, the QMT and QLL can use the address of a packet’s FDB to represent the packet’s address. Similar to the PLL, the QLL stores all of the packets from the same queue in the form of a singly linked list. The difference is that the QLL stores the Next Packet Address (NPA) instead of NDBA.

The QMT stores and updates the address of the first packet and the last packet of each queue. Please notice that two packets with the same QID but different QGIDs, belong to different queues in the QMT. Each time the PLL receives an FDB address from the DSS, it forwards this address to the QMT. Then, the QMT needs to judge whether this is the first arriving packet of an empty queue. If yes, the related QID, QGID, and PL of this packet are enqueued to QGWFQS by the QMT immediately. If not, the QMT updates the address of the last packet of this queue and sends related information to QLL to help it update the singly linked list.

When the QMT receives the QID and QGID from QGWFQS, the first packet of this queue should be dequeued and read out from the SPB. The address of the first packet of this queue is then sent to the PLL as the address of a packet’s FDB. Soon, the whole packet will be read out from the SPB. At the same time, the QMT asks the QLL for the address of this first packet’s next packet. If this queue remains more than one packet, QLL will answer the address. The QMT then updates the answered address as the address of the first packet of this queue from this time on. The QMT also enqueues this queue again to QGWFQS. Back to the asking, if this queue remains only one packet, the QLL will answer None. The QMT records that this queue is empty.

### 3.2. Tag Calculator

The main function of the Tag Calculator is to compute the finish tag (FT) according to the receiving QID, QGID, and PL of the packet from the SPB. Then, the FT, along with the corresponding QID and QGID, is transmitted to Queue Select.

#### 3.2.1. Tag Calculation Algorithm

In order to deal with the remainder obtained from each calculation of integer division, we refer to the concept of token bucket and introduce an attribute called Queue Token (QT) to each queue. Once the finish tag of $p_{i}^{j}$ is computed, the QT is subsequently updated accordingly. The updated QT is denoted as $T_{i}^{j}$. The algorithm of the Tag Calculator can be expressed as Equations (6)–(9).

(6) $$S\left(p_{i}^{j}\right)=max\left\{v\left(A\left(p_{i}^{j}\right)\right),F\left(p_{i}^{j-1}\right)\right\},j\geq 1,$$

(7) $$F\left(p_{i}^{j}\right)=\left\{\begin{array}{cc} S\left(p_{i}^{j}\right)+\left\lceil \frac{l_{i}^{j}-T_{i}^{j-1}}{w_{i}}\right\rceil & ,\;\mathrm{if}\;j\geq 1\\ 0 & ,\;\mathrm{if}\;j=0 \end{array}\right.$$

(8) $$T_{i}^{j}=\left\lceil \frac{l_{i}^{j}-T_{i}^{j-1}}{w_{i}}\right\rceil w_{i}-\left(l_{i}^{j}-T_{i}^{j-1}\right)$$

(9) $$v\left(t\right)=F_{l}^{k},\;s_{l}^{k}<t\leq d_{l}^{k},$$

The value of the QT actually represents the excess packet length that is accounted for during the calculation of the finish tag of the previous packet of the queue to ensure integer division without a remainder. Therefore, when computing the finish tag of the next packet of the queue, it is necessary to deduct this value from the packet length in advance.

#### 3.2.2. Tag Calculator Design

**Figure 3:**
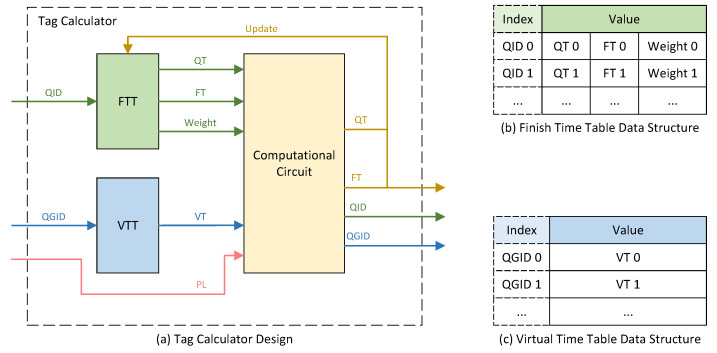
Tag Calculator design. (**a**) Tag Calculator structure. (**b**) Finish time table data structure. (**c**) Virtual time table data structure. FTT: Finish time table. VTT: Virtual time table. QT: Queue Token.

The detailed design of the Tag Calculator module is illustrated in Figure 3a. The module comprises two tables, namely the Finish Tag Table (FTT) and the Virtual Time Table (VTT), along with a Computational Circuit. Upon receiving an enqueue request, the QID is used as the address to query the FTT, retrieving the previously calculated QT and FT of the last packet enqueued of the queue, as well as the Weight of the queue. Similarly, using the QGID as the address, the VTT is queried to obtain the Virtual Time (VT) of the queue group. The computational circuit utilizes the retrieved values from both tables, along with the PL provided during the enqueue request, to calculate the FT and QT of the current packet, and subsequently updates the FTT.

The data structure of the FTT is depicted in Figure 3b. The number of entries in the FTT is equal to the product of the number of queues per queue group and the number of queue groups. Each FTT entry consists of the QT, FT, and Weight. The QT and FT are updated each time a new FT of the queue is computed. The Weight is user-configurable, representing the Weight assigned to the respective queue.

Figure 3c illustrates the data structure of the VTT. The depth of the VTT is equal to the number of queue groups. Each VTT entry caches the VT of the corresponding queue group. When a packet from a particular queue group is scheduled and is departing from the queue group, the FT of this packet serves as the VT for that queue group in the subsequent period of time, and the corresponding entry in the VTT is updated accordingly.

**Figure 4:**
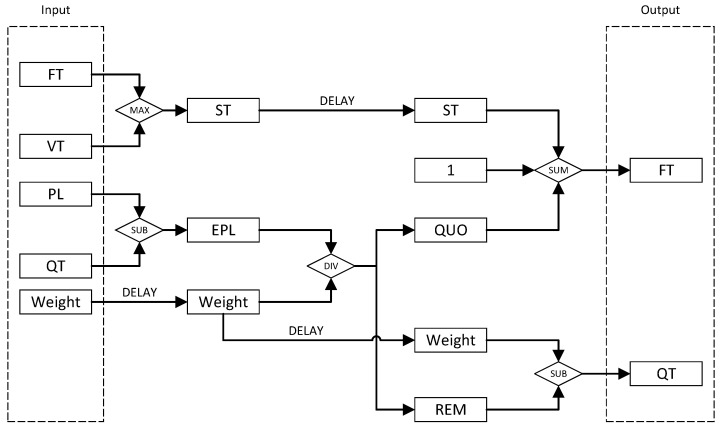
Computational circuit. FT: finish time. VT: virtual time. ST: start time. PL: packet length. EPL: edited packet length. QT: queue token. QUO: quotient. REM: remainder.

The design of the Computational Circuit is depicted in Figure 4. The computation of the FT and QT involves one sum operation, two subtraction operations, one comparison operation, and one integer division operation. This circuit is pipelined, allowing the respective values of a queue to be inputted into the circuit in each clock cycle. After a fixed period of time, the circuit computes the FT of the head packet of that queue. Due to the inherent clock cycle delay in FPGA-based computations, certain values are delayed by a corresponding number of cycles to synchronize with the completion of other related values.

In the first clock cycle, the values of the inputted FT and VT pass through a comparison circuit, wherein the larger value is selected. The ST is then updated according to this value. Simultaneously, the value of the QT is deducted from the value of the PL to obtain the Edited Packet Length (EPL). The purpose of this operation is to eliminate the impact on fairness caused by the integer division not resulting in an exact quotient when calculating the finish tag of the previous packet within the same queue. Subsequently, the obtained EPL is divided by the delayed Weight, resulting in a quotient and a remainder, denoted as QUO and REM, respectively. This operation may require a time duration exceeding a single clock cycle. Finally, the updated value of the FT is obtained by adding the value of the ST to QUO and incrementing it by one. The additional increment is necessary because, as stated in the formula, the quotient is a ceiling to the nearest larger integer. Simultaneously, the updated value of the QT is derived by subtracting the REM from Weight. The newly obtained values of FT and QT serve as outputs of the circuit, which are used to update the corresponding entries in the FTT. The FT is also transmitted to the Queue Select module.

### 3.3. Queue Select

The Queue Select module serves two primary functions. Firstly, it receives the FT from the Tag Calculator module and caches it at the corresponding position based on the concurrently received QGID and QID. Secondly, upon receiving the signal indicating an idle queue group (IDLE QGID), it retrieves the FTs of all enqueued queues belonging to that queue group. Subsequently, the retrieved FTs are subjected to a comparison operation. Finally, the FT with the minimum value is selected, and its corresponding QID and QGID are sent as dequeue information to the SPB.

**Figure 5:**
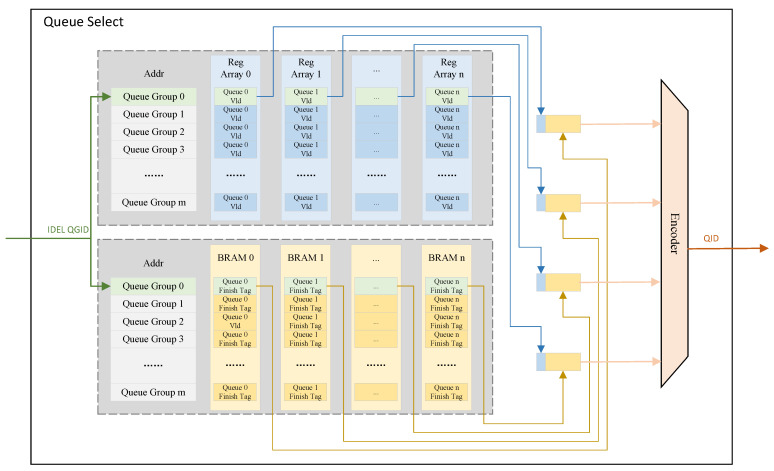
Queue select design.

The detailed design of the Queue Select module is illustrated in Figure 5. *N* BRAM-based tables, each with a depth of *M*, are utilized for caching FTs. *N* Register Arrays, each with a depth of *M*, are employed to indicate the validity of the FTs cached at the respective positions. Additionally, the Queue Select module includes an Encoder, which aims to identify the minimum value among *N* input numbers within a fixed time and output the corresponding channel ID associated with that number. The channel ID is regarded as the QID of the minimum valid FT.

When the FT is input from the Tag Calculator module, the corresponding BRAM-based table is selected based on the concurrently input QID. The value of the FT is written into this table, and the address where the value is written is indicated by the concurrently input QGID. Simultaneously, the corresponding Register Array is chosen based on the QID. The Vld register associated with the QGID in this Register Array is then asserted.

**Figure 6:**
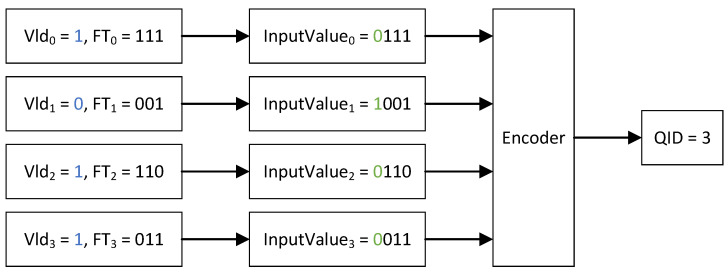
Select the queue with the minimum finish tag among all valid finish tags.

When the IDLE QGID is received, all FTs cached at the address indicated by the QGID are read out from all the BRAM-based tables. Similarly, the values of Vld registers at the corresponding positions in all Register Arrays are also read out. To prevent invalid FTs from influencing the encoding result, the respective valid bits are inverted and concatenated to the leftmost side of the highest bit of each read-out FT. Thus, each of the values that are pushed into the Encoder is 1 bit wider than the FT. The Encoder is designed as a binary tree. The input values are the leaf nodes of this tree. Every clock period of FPGA, two child nodes of the same parent node are compared and the parent node is updated with the smaller value. Finally, the root node is updated with the smallest value among all the input values. There are no valid FTs if the highest bit of the minimum value is one, and the Encoder outputs nothing. Otherwise, the FT corresponding to the ultimately found minimum value is guaranteed to be the smallest FT among all valid FTs read from BRAM-based tables. The Encoder outputs the corresponding channel ID as the QID. Figure 6 is an example to illustrate why the minimum valid FT among all the FTs can be found. If the Vld signal of the queue is not asserted, then the value pushed into the Encoder has the highest bit of 1, and this value is greater than any other values with a Vld signal equal to 1. As a result, the input value of channel 3 is less than the one from channel 1, even if FT3 is greater than FT1.

## 4. Implementation and Result

**Figure 7:**
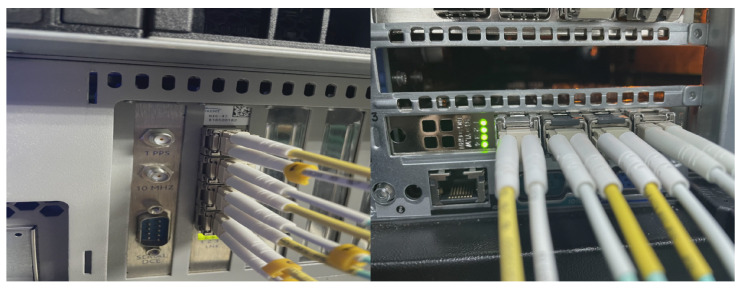
Hardware environment. On the (**left side**), the ports of the Spirent C50 are shown, while on the (**right side**), the ports of the FPGA board are displayed. Each pair of these ports is connected by optical fiber.

We developed the proposed QGWFQS architecture in Verilog. Modesim SE-64 2020.4 was used for simulation during the development. We also usde Vivado 2020.2 to synthesize the project and finally generated the bit file, which was downloaded to a Xilinx Kintex-7 series xc7k325tffg900-2L FPGA deployed on a Dell R740 commodity server. As shown in Figure 7, each port of FPGA is connected to Spirent C50 through optical fiber in order to test network performance. The resource consumption and performance are evaluated.

The implemented QGWFQS supports 512 queue groups, each of which consists of 32 queues. The total number of scheduled queues is 16 k. The compilation results indicate that both the Tag Calculator and Queue Select modules achieve a clock frequency of 200 MHz. Due to the pipeline design of the entire system, the QGWFQS is capable of scheduling up to 200 million packets per second. As a comparison, a fully hardware-based work [12] can schedule packets with an average length of 80 bytes at 12.8 Gbps. The scheduling capability of the compared work is about 16 million packets per second. The performance of the QGWFQS to schedule an individual queue is dependent on the number of clock cycles it experiences, from each enqueue operation into the QGWFQS, to each dequeue operation from the QGWFQS. With a delay of 16 cycles, the QGWFQS can schedule a maximum of 12.5 million packets per second from a single queue.

### 4.1. Hardware Resource Evaluation

The resource utilization of the Tag Calculator circuit is presented in Table 1, alongside the resource utilization of the method proposed by McKillen [6], which serves as a basis for comparison. Our approach supports tag computation for 16 k queues across 512 queue groups, while the comparative work needs to be instantiated 512 times to achieve a similar effect. The total resource consumption of the comparative work is 512 times the resource consumption of a single instantiation, resulting in a higher total resource utilization.

It is noteworthy that the implemented Tag Calculator circuit also incorporates additional functionalities such as initialization and Weight reconfiguration. The resource consumption of the required LUTs and FFs for implementing these functionalities is also included in the analysis. In contrast, the resource consumption listed for the comparative work only considers part of the computational circuits, while other consumption is not taken into account.

**Figure 8:**
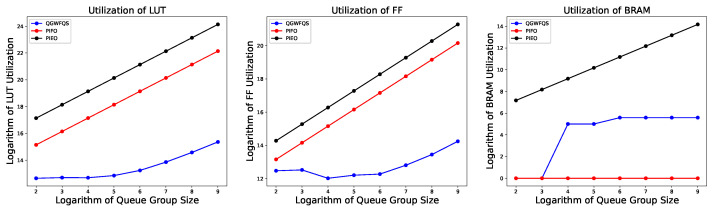
Resource utilization of Queue Select.

When the number of supported queue groups changes, the resource utilization of the Queue Select module is depicted in Figure 8. For ease of visualization, both the horizontal and vertical axes are logarithmically scaled. It is observed that when the number of supported queue groups is relatively small, our approach utilizes LUTRAM to cache the FT, resulting in zero consumption of BRAM resources. However, as the number of supported queue groups increases, our approach starts utilizing BRAM as the storage resource for the FT. The utilization of LUT and FF resources in our approach also exhibits a linear relationship with the number of supported queue groups, as we employ a Register Array to store the signals indicating the validity of the FT.

We compared our work with PIFO and PIEO as reference models. Our work utilizes a single Encoder across 512 queue groups. Consequently, compared with existing methods, our proposed method demonstrates an absolute advantage in terms of resource consumption. Existing methods can easily implement a scheduler capable of scheduling 32 queues on FPGA. However, as the number of queue groups increases and the number of schedulers grows, the linear growth in resource consumption renders these methods infeasible for implementation on FPGA.

### 4.2. Fairness of Bandwidth Allocation

We evaluated both short-term and long-term bandwidth allocation fairness. The fairness of the short-term allocation was assessed using the Normalized Fairness Metric (NFM) [20]. For an interval of duration τ, denote the actual number of bytes served by each queue as $Sch_{i}$; then, the calculation algorithm for the NFM is found in Equation (10).

(10) $$NFM\left(\tau \right)=\max\left(\frac{Sch_{i}}{w_{i}}-\frac{Sch_{j}}{w_{j}}\right)*\frac{\sum w_{i}}{C(t)*\tau },\;\mathrm{for}\;i,j=1,2,\ldots ,n,\;\mathrm{and}\;t\in \tau ,$$

**Figure 9:**
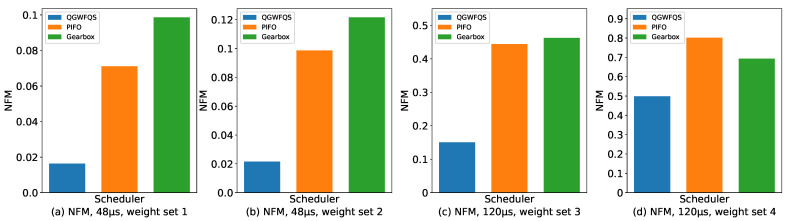
Normalized Fairness Metric.

The Weight sets we configured for evaluation consisted of four variations, as shown in Table 2. The corresponding NFM values obtained for each Weight set are presented in Figure 9.

**Figure 10:**
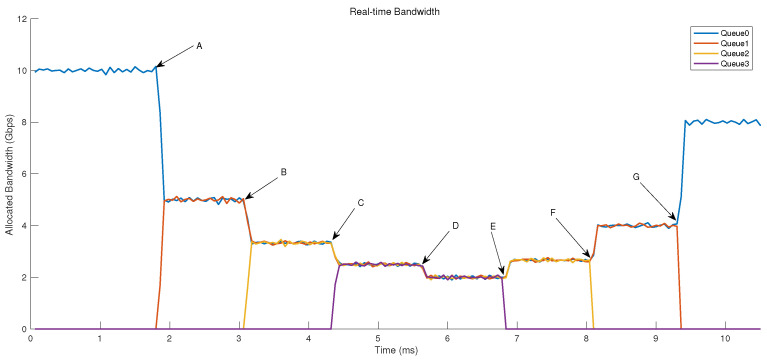
Allocated bandwidth. From A to C, queues become active successively. At D, total bandwidth changes. From E to G, queues become inactive successively.

We evaluated the long-term bandwidth allocation fairness in an actual network, and the results are depicted in Figure 10. We designed a scenario where there are four queues, each of which is configured with the same Weight. Initially, the total bandwidth allocated to the queue group was 10 Gbps. As only one queue was active at the start, it could fully utilize the entire bandwidth of the queue group. At time A, another queue became active, and both queues were allocated 5 Gbps of bandwidth each. At times B and C, the third and fourth queues joined, and all queues continued to share the total 10 Gbps bandwidth equally.

At time D, the total bandwidth of the queue group suddenly decreased from 10 Gbps to 8 Gbps. Consequently, the bandwidth allocated to each queue decreased from 2.5 Gbps to 2 Gbps. The fairness of bandwidth allocation was maintained despite the variation in the link rate of the queue group. Starting from time E, the number of active queues began to decrease. Regardless of the number of remaining queues, they could evenly share the 8 Gbps bandwidth. After time G, only one active queue remained, which started to fully utilize the available bandwidth of the queue group from that moment.

## 5. Conclusions

We propose a queue-group-based Weighted Fair Queuing scheduler capable of supporting up to 16 k queues across 512 queue groups, providing bandwidth allocation services. Experimental results demonstrate that the proposed scheduler exhibits advantages in terms of resource efficiency and fairness. Furthermore, bandwidth allocation fairness is still maintained even when the total bandwidth of the queue groups changes.

## Figures and Tables

**Table 1:** Resource utilization of Tag Calculator.

	Our Work	Comparative Work [6]
LUTs	1302	284,672
Flip Flops	883	338,944
BRAMs	45.5	768

**Table 2:** Weight sets.

Weight Set	Weight
Weight Set 1	1:1:1:1
Weight Set 2	2:2:1:1
Weight Set 3	50:50:1:1
Weight Set 4	100:100:1:1

## Data Availability

All the necessary data are included in the article.
